# Long-term prognostic value of microvascular obstruction by cardiac magnetic resonance in ST-segment elevation myocardial infarction

**DOI:** 10.1371/journal.pone.0344442

**Published:** 2026-03-06

**Authors:** Raquel P. Amier, Casper W. H. Beijnink, José F. Rodriguez Palomares, Martijn W. Smulders, Gonzalo Pizarro Sánchez, Rodrigo Fernández-Jiménez, Filipa X. Valente, Albert C. van Rossum, Niels van Royen, Borja Ibañez, Robin Nijveldt

**Affiliations:** 1 Department of Cardiology, Amsterdam University Medical Center, Amsterdam, the Netherlands; 2 Department of Cardiology, Radboud University Medical Center, Nijmegen, the Netherlands; 3 Department of Cardiology, Hospital Universitario Vall d’Hebron, Vall Hebron Institut de Recerca, Universitat Autònoma de Barcelona, Barcelona, Spain; 4 Centro de Investigación Biomédica en Red (CIBER)-CV, Madrid, Spain; 5 Department of Cardiology, Cardiovascular Research Institute Maastricht (CARIM), Maastricht University Medical Centre+ (MUMC+), Maastricht, the Netherlands; 6 Centro Nacional de Investigaciones Cardiovasculares (CNIC), Madrid, Spain; 7 Department of Cardiology, IIS Fundación Jiménez Díaz Hospital, Madrid, Spain; 8 Netherlands Heart Institute, Utrecht, the Netherlands; Scuola Superiore Sant'Anna, ITALY

## Abstract

**Aims:**

Microvascular obstruction (MVO) portends a higher risk of remodelling and adverse events following ST-segment elevation myocardial infarction (STEMI). However, data regarding the implications of MVO in STEMI beyond five years of follow-up are scarce.

**Methods & Results:**

This is a pooled analysis of three observational studies including 876 prospectively enrolled reperfused first STEMI patients, who underwent cardiac magnetic resonance imaging with late gadolinium enhancement (LGE), between 2003–2019. Median follow-up duration was 6.3 (IQR 3.6–9.3) years. The primary outcome was all-cause mortality. The secondary outcome was a combined endpoint of all-cause mortality and recurrent ischemic events (i.e., myocardial infarction or stroke). We performed Cox regression analyses with a time-dependent covariate.

The study population consisted of 876 patients, mean age 59 years ± 12, 720 men (82%). MVO was present in 499 patients (58%). The presence of MVO was independently associated with all-cause mortality up to six years post-STEMI (Hazard Ratio [HR] 2.23, 95% CI 1.09–4.57, p = 0.029), but not after six years post-STEMI (HR 0.98, 95% CI 0.45–2.12, p = 0.958). Presence of MVO was not significantly associated with a combined endpoint of all-cause mortality and recurrent ischemic events before or after six years of follow-up (HR 1.27, 95% CI 0.81–1.99, p = 0.294 and HR 0.68, 95% CI 0.35–1.31, p = 0.244, respectively).

**Conclusions:**

In STEMI patients, the presence of MVO by cardiac magnetic resonance imaging is associated with a more than two-fold higher risk of all-cause mortality up to six years after the index event. This relation seems to dissipate beyond this time period.

## Introduction

Despite swift epicardial coronary reperfusion, a substantial proportion (50–66%) of patients with ST-segment elevation myocardial infarction (STEMI) have signs of microvascular obstruction (MVO) at cardiac magnetic resonance imaging (CMR) [1–3]. MVO occurs more often in patients with larger infarctions and those with more cardiovascular co-morbidity, and has become an important predictor for adverse cardiac remodelling and cardiovascular events [4,5].

A vast research field has emerged, aimed at understanding the pathophysiological mechanisms underlying MVO and therapies for the prevention of MVO. It is the current consensus that MVO should be assessed in clinical STEMI trials as a secondary outcome, serving as a surrogate marker (or proxy) for clinical outcome, but that long-term effects of MVO have not yet been studied sufficiently to support its use as a primary outcome [6]. The use of MVO to guide tailored therapy strategies in clinical practice remains under debate.

The prognostic impact of MVO in STEMI patients is well-studied, mostly at one to two years after the index event. Meta-analyses of these studies show a strong relation with heart failure, recurrent myocardial infarction, and death [3,7]. The few studies that have investigated the prognostic impact of MVO at long-term follow-up, confirm that MVO is a strong prognosticator for heart failure and revascularization up to five years post-STEMI [8,9]. Although an association between the presence of early MVO and cardiovascular mortality has been demonstrated on the univariable level, no study has yet corrected for infarct size and left ventricular ejection fraction [10].

The current study evaluates the relation of MVO and all-cause mortality in a cohort of patients with first STEMI treated by primary percutaneous coronary intervention (PCI) with the longest follow-up to date, with correction for important covariables. In addition, we evaluate the relation of MVO with a composite of all-cause mortality and ischemic events, i.e., recurrent myocardial infarction (MI) and stroke.

## Materials and methods

### Study population

**Fig 1:**
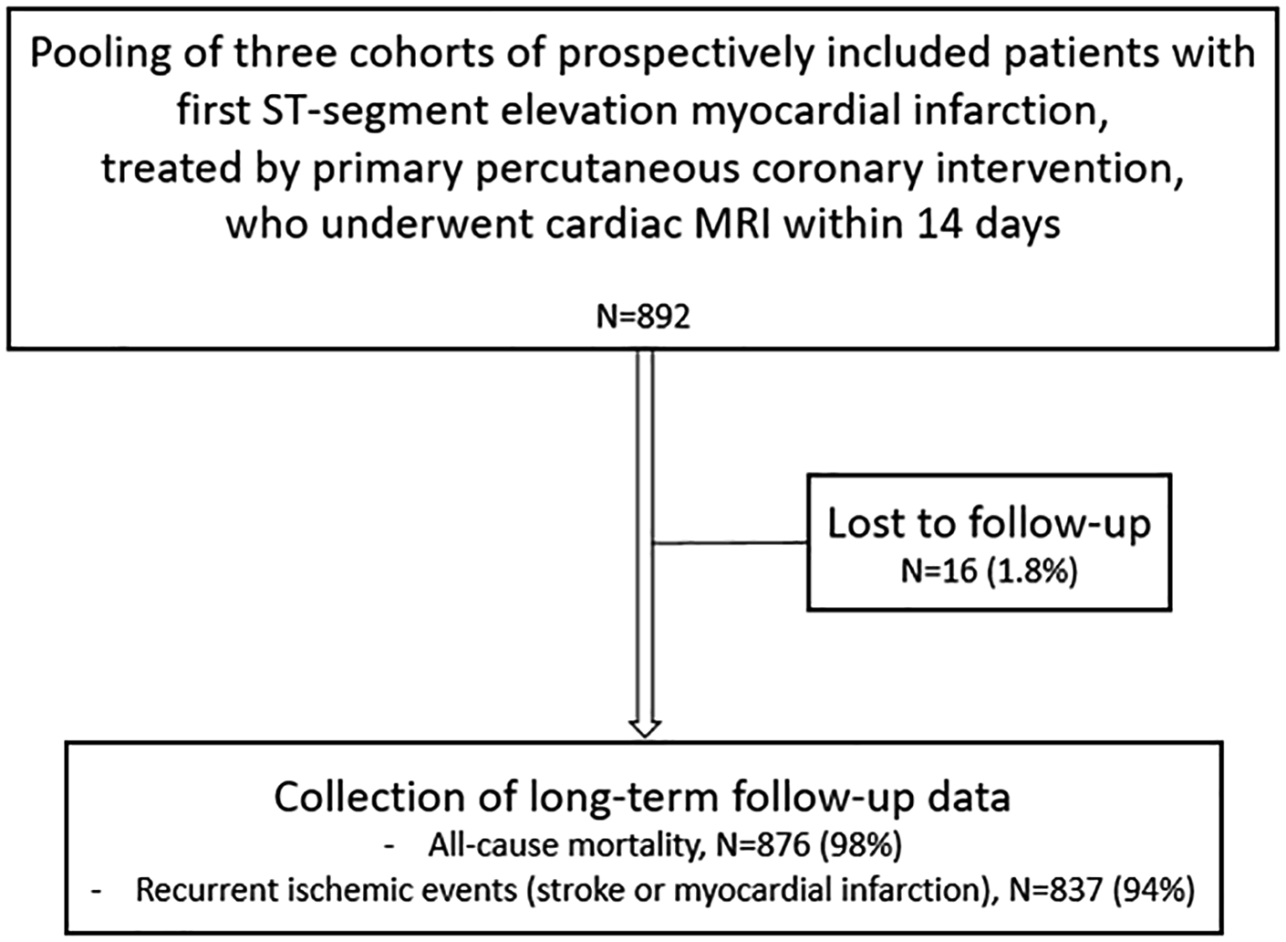
Study flow chart.

We performed a pooled analysis of three clinical studies including first STEMI patients (total n = 892) between 2003–2019 who underwent CMR imaging within 14 days after hospital admission. Clinical endpoints were retrospectively collected. Sixteen patients (1.8%) were lost to follow-up (Fig 1).The remaining 876 patients had been prospectively enrolled in one of the following studies; i) the METOCARD-CNIC trial [11] that assessed the effect of intravenous metoprolol before primary PCI in STEMI patients (n = 199 [23%]), ii) an existing database of prospectively enrolled first acute MI patients at two University Medical Centers in the Netherlands (n = 245 [28%] and n = 88 [10%]), and iii) a prospective registry study conducted at a University Medical Center in Spain (n = 344 [39%]), results from which have been previously published [12–14]. All patients have been previously reported in these studies and the current study adds analyses of prognostic value of microvascular obstruction on clinical outcome and extends follow-up duration, in a pooled patient population. The Institutional Review Board of all three centers gave permission for the use of data for future research purposes and all patients had previously consented to the use of their data for clinical research and for follow-up.

### Clinical follow-up and clinical outcomes

The primary outcome was all-cause mortality, which was retrieved from public databases (Historal Electrònic de Salut in Catalunya, Spain, and Municipal Civil Registry in the Netherlands) and for patients included from the METOCARD-CNIC trial from the medical record or a death certificate. The secondary outcome was a composite of all-cause mortality and ischemic events (stroke or recurrent MI). Clinical events were collected during direct patient contact, a telephone interview and from medical records. Follow-up was performed between 2015 and 2021, resulting in a median duration of 6.3 (interquartile range [IQR] 3.6–9.3) years. The data were accessed for research purposes between 01-06-2022 and 01-10-2022. Mortality data were available for 876 (98%) patients and outcome data for the composite endpoint for 837 patients (94%).

### CMR imaging

All patients underwent CMR imaging at a median of 5 (IQR 4–7) days after hospital admission. The CMR protocol comprised balanced steady-state free precession cine images on clinical 1.5 T MRI scanners in standard long axis and short axis views. Gadolinium-based contrast agents were administered at a dosage of 0.1–0.2 mmol/kg, followed by late gadolinium enhancement (LGE) image acquisition 10–12 minutes afterwards.

### CMR image analysis

**Fig 2:**
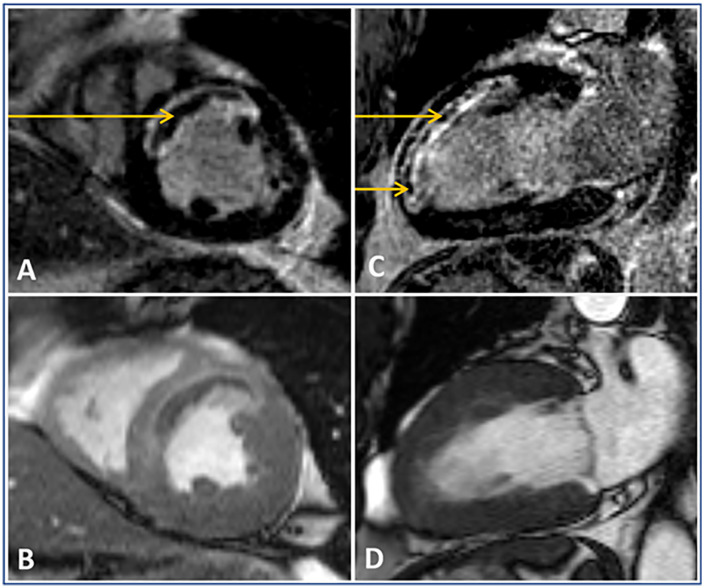
Example of microvascular obstruction after anterior ST-segment elevation myocardial infarction. Panel A shows a short-axis late gadolinium enhancement (LGE) cardiac magnetic resonance (CMR) image of an anterior ST-segment elevation myocardial infarction (STEMI) with microvascular obstruction (MVO). Panel B is the corresponding end-systolic cine frame. Panel C is the long-axis 2-chamberview LGE image of the same patient, showing MVO, and panel D is the corresponding cine frame. MVO is arrowed in all LGE CMR frames. Abbreviations: CMR: Cardiac Magnetic Resonance, LGE: Late gadolinium enhancement, MVO: Microvascular obstruction, STEMI: ST-segment elevation myocardial infarction.

LV volumes and function were determined on the short axis cine images by manual contour tracing. LV end-diastolic volume and end-systolic volume were obtained from cine images. Infarct size was quantified manually by planimetry on the short axis LGE images [15] or determined using the full-width at half maximum method [11,12], and expressed in grams. The LV ejection fraction (EF) was calculated as (LV end-diastolic volume – LV end-systolic volume)/ LV end-diastolic volume * 100%. MVO was defined visually as the presence of a relatively hypo-enhanced core surrounded by hyperenhanced myocardium on the LGE images. See Fig 2 for an example of MVO. CMR image analyses were performed by a trained cardiology research fellow (RA, with two years of experience) and controlled by a cardiologist specialized in cardiac imaging (RN, with 12 years of experience) for 532 patients (from databases i and ii); and by a cardiologist specialized in cardiac imaging (FV, with 8 years of experience for 344 patients (from database iii), with consensus in case of doubt with a cardiologist specialized in cardiac imaging (JRP, with 13 years of experience). All image analyses were performed with anonymized data, with readers blinded to clinical data, using dedicated off-line software (QMassMR version 7.6, Medis, Leiden, the Netherlands; or Cvi42, Circle Cardiovascular Imaging Inc., Calgary, Alberta, Canada).

### Statistical methods

Dichotomous variables are shown as a number with percentage and compared using the Chi-squared test. Continuous variables are depicted as mean ± standard deviations (SD) and compared with the Student T test. In case of non-normal distribution, variables were depicted as median with IQR and compared using the Mann-Whitney U test. We performed Cox regression analysis to assess the relation between presence of MVO and clinical outcome. For the composite endpoint, events were prioritized according to severity, with death > stroke > MI in cases with multiple events.

In standard Cox regression analyses of MVO with all-cause mortality and recurrent ischemic events, the proportional hazards assumption was not met and therefore Cox regression analyses were performed with a time-dependent covariate, set at t = 6 years (p = 0.047 for the primary endpoint [all-cause mortality] and p = 0.046 for the secondary endpoint [all-cause mortality and recurrent ischemic events], indicating the significant difference in HR up to six years follow-up vs. after six years follow-up). This was supported by visual interpretation of the Kaplan Meier survival curves, and this was the cutoff with the highest statistical significance for the time-dependent covariate. For 850 patients (97%), covariable data was complete. Following the univariable analysis, these patients were included in two multivariable Cox regression models. Model 1 included correction for clinical covariables only, i.e., age, sex, past or present smoking, history of diabetes mellitus, history of hypertension, and LAD culprit artery. Model 2 included imaging characteristics, i.e., infarct size and LV ejection fraction, in addition to the clinical covariable. All analyses were performed using SPSS (IBM® SPSS ® Statistics, Version 25).

## Results

The baseline characteristics are shown in Table 1. Briefly, a total of 876 patients were included, 59 ± 12 years old, and 82% were male. Patients with MVO were more likely to have an anterior STEMI than those without (63% vs. 46%, p < 0.001). Secondly, patients with MVO had a significantly lower LV ejection fraction (44 ± 9% vs. 53 ± 9%, p < 0.001) and larger infarct size (32 [IQR 22–45] gram vs. 14 [6–24] gram, p < 0.001) than those without MVO. The baseline characteristics of patients who were lost to follow-up are shown in Supplemental Table 1. In summary, these patients were slightly older (53 ± 12 years vs. 59 ± 12 years, p = 0.04) and had slightly larger LV end-diastolic volume (189 ± 35 vs. 165 ± 41 mL, p = 0.009) but ejection fraction was similar (43 ± 11% vs. 48 ± 10%, p = 0.07).

**Table 1:** Patient characteristics.

	All patients (N = 876)	MVO (n = 499, 58%)	No MVO (n = 377, 42%)	p-value
Demographics				
Age, years (SD)	59 ± 12	59 ± 12	60 ± 11	0.08
Male, n (%)	720 (82)	429 (86)	291 (77)	<0.001
Follow-up duration, yrs	6.3 (3.6-9.3)	7.8 (3.8-9.5)	5.3 (3.3-9.0)	<0.001
Cardiovascular risk factors				
Smoking, n (%)	567 (66)	335 (69)	232 (63)	0.07
Hypertension, n (%)	340 (39)	181 (37)	159 (43)	0.08
Dyslipidemia, n (%)	293 (35)	160 (33)	133 (37)	0.28
Diabetes mellitus, n (%)	113 (13)	63 (13)	50 (13)	0.80
Coronary angiography				
Anterior STEMI, n (%)	482 (56)	311 (63)	171 (46)	<0.001
CMR parameters				
CMR, day	5 (456–7)	5 (456–7)	5 (456–7)	0.25
LV end-diastolic volume, ml	165 ± 41	173 ± 41	154 ± 38	<0.001
LV end-systolic volume, ml	88 ± 31	97 ± 31	75 ± 25	<0.001
LV ejection fraction (%)	48 ± 10	44 ± 9	53 ± 9	<0.001
Infarct size (g)	24 (13-37)	32 (22-45)	14 (6-24)	<0.001
Infarct size (%LV)	21 (12-31)	28 (20-37)	12 (6–21)	<0.001

Abbreviations: CMR: Cardiac Magnetic Resonance, LV: Left ventricle, MVO: Microvascular obstruction, STEMI: ST-segment elevation myocardial infarction.

### Event rates during long-term follow-up

During the median follow-up period of 6.3 (IQR 3.6–9.3) years, 123 patients (14%) experienced a clinical event. Sixty-seven patients died (8%), 20 patients suffered stroke (2%) and 35 (4%) had a recurrent MI (Table 2).

**Table 2:** Event rates during follow-up.

	Total study (N = 876)	MVO (n = 499)	No MVO (n = 377)
Death, n (%)	67 (7.6)	42 (8.4)	25 (6.6)
Stroke, n (%)	20 (2.3)	7 (1.4)	13 (3.4)
Recurrent myocardial infarction, n (%)	35 (4.0)	16 (3.2)	19 (5.0)
Total clinical events, n (%)	123 (14.0)	65 (13.0)	57 (15.1)

Abbreviations: MVO: Microvascular obstruction.

### Relation of MVO with all-cause mortality

**Fig 3:**
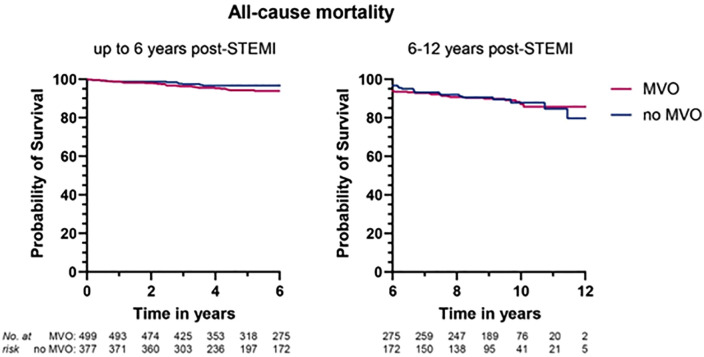
Uncorrected survival curves in patients with and without microvascular obstruction. Kaplan Meier graphs showing uncorrected survival curves for all-cause mortality in those with and without microvascular obstruction. Abbreviations; MVO: Microvascular obstruction, STEMI: ST-segment elevation myocardial infarction.

Uncorrected Kaplan Meier survival curves are shown in Fig 3. Regression analyses are shown in Tables 3 and 4. The presence of MVO was significantly associated with all-cause mortality up to six years post-STEMI (HR = 2.23, 95% CI = 1.09–4.57), independent from clinical characteristics. From six years and beyond, MVO was no longer a significant predictor of mortality (HR = 0.98, 95% CI = 0.45–2.12). In the second model (corrected for clinical and imaging characteristics), MVO was not significantly associated with mortality (HR = 1.53, 95% CI 0.66–3.54 up to six years post-STEMI and HR = 0.62, 95% CI 0.26–1.50, from six years and beyond, Table 3). Age was the only significant predictor in this model (HR = 1.09, 95% CI = 1.06–1.12, per year increase). There was no association between sex and all-cause mortality.

**Table 3:** Cox regression analyses for MVO and all-cause mortality.

	Model 1		Model 2	
	HR (95% CI)	p-value	HR (95% CI)	p-value
MVO, ≤ 6 years	2.23 (1.09-4.57)	0.029	1.53 (0.66-3.54)	0.32
MVO, > 6 years	0.98 (0.45-2.12)	0.96	0.62 (0.26-1.50)	0.29
Age	1.08 (1.05-1.11)	<0.001	1.09 (1.06-1.12)	<0.001
Sex (male)	1.06 (0.58-1.92)	0.85	0.78 (0.40-1.54)	0.48
Smoking	1.08 (0.62-1.89)	0.79	1.03 (0.57-1.85)	0.93
Diabetes	1.52 (0.84-2.74)	0.17	1.53 (0.84-2.79)	0.17
Hypertension	1.36 (0.81-2.29)	0.24	1.65 (0.96-2.86)	0.07
LAD culprit artery	1.08 (0.63-1.85)	0.78	0.76 (0.42-1.37)	0.36
Infarct size (g)	–	–	1.00 (0.99-1.02)	0.70
LV ejection fraction	–	–	0.97 (0.94-1.00)	0.08

Abbreviations: HR: Hazard ratio, CI: Confidence interval, LAD: Left anterior descending coronary artery, LV: Left ventricle, MVO: Microvascular obstruction.

**Table 4:** Cox regression analyses for MVO and combined death, stroke, and myocardial infarction.

	Model 1		Model 2	
	HR (95% CI)	p-value	HR (95% CI)	p-value
MVO, ≤ 6 years	1.27 (0.81-1.99)	0.29	1.22 (0.71-2.09)	0.48
MVO, > 6 years	0.68 (0.35-1.31)	0.25	0.55 (0.26-1.13)	0.10
Age	1.04 (1.02-1.05)	<0.001	1.04 (1.02-1.06)	<0.001
Sex (male)	1.00 (0.63-1.58)	>0.99	0.81 (0.49-1.36)	0.81
Smoking	1.02 (0.67-1.54)	0.94	0.99 (0.64-1.52)	0.96
Diabetes	1.29 (0.81-2.06)	0.28	1.27 (0.79-2.04)	0.33
Hypertension	1.38 (0.94-2.02)	0.10	1.39 (0.94-2.08)	0.10
LAD culprit artery	1.01 (0.69-1.49)	0.95	0.90 (0.60-1.37)	0.90
Infarct size	–	–	1.00 (0.99-1.02)	0.81
LV ejection fraction	–	–	0.99 (0.97-1.02)	0.49

Abbreviations: HR: Hazard ratio, CI: Confidence interval, LAD: Left anterior descending coronary artery, LV: Left ventricle, MVO: Microvascular obstruction.

### Relation of MVO with all-cause mortality and recurrent ischemic events

The presence of MVO was not significantly associated with a composite endpoint of all-cause mortality and recurrent ischemic events (HR = 1.27, 95% CI 0.81–1.99 up to six years post-STEMI and HR = 0.68, 95% CI 0.35–1.31 from six years and beyond). In the second model (correcting for clinical and imaging markers), MVO did not predict all-cause mortality and recurrent ischemic events up to 6 years (HR = 1.22, 95% CI 0.71–2.09) or thereafter (HR = 0.55, 95% CI 0.26–1.13) either, see Table 4.

## Discussion

In this pooled analysis, we assessed the long-term prognostic value of MVO by LGE-CMR in patients presenting with first STEMI treated by primary PCI. The main finding is that the presence of MVO is significantly associated with all-cause mortality up to six years post-STEMI, but not beyond that time. This prognostic effect is independent from cardiovascular risk factors, but not independent from infarct size and LV ejection fraction. The presence of MVO was not significantly associated with a combined endpoint of mortality and recurrent ischemic events.

In line with previous studies, patients with MVO had a larger infarct size [3] and lower LV ejection fraction [16] than those without MVO. A strong relation between MVO and increased risk of mortality and heart failure has been consistently established in the first two years post-STEMI, largely independent from infarct size and LV ejection fraction [3,5,17,18]. At two to three years of follow-up, MVO has been associated with MACE, importantly driven by heart failure and reinfarction (62–81% of events) [19,20]. While there is a clear link between MVO and heart failure at longer follow-up duration as well, the relation with mortality beyond two years post-STEMI is less apparent from the currently available literature. In a previous study, MVO was associated with heart failure and revascularization, but not with mortality, at a median of four years of follow-up in 77 STEMI patients [9]. In another larger study in 811 STEMI patients, MVO was associated with MACE at a median follow-up of 5.5 years, largely determined by higher rates in heart failure (21 vs 3%, compared to mortality rates of 7 vs 4% in those with and without MVO, respectively) [8]. Recently, Fisher et al.[10] assessed the cause of death in a cohort of 475 patients. Among patients with MVO, the cause of death was most frequently cardiovascular in nature, whereas cardiovascular death was rare in patients without MVO.

In our study, we observed an increased mortality risk in STEMI patients with MVO, which seems to dissipate from six years after the index event. In addition, the presence of MVO did not hold additional predictive value over LVEF and infarct size up to six years follow-up with regard to all-cause mortality. On the other hand, given the consistent association between MVO and heart failure, irrespective of LVEF and infarct size [8,9], MVO retains some value as a post-infarct prognosticator. Nevertheless, our results do not support the use of MVO as a surrogate primary outcome in STEMI trials.

In addition, the presence of MVO was not associated with a combined endpoint of all-cause mortality and recurrent ischemic events. While mortality data were largely obtained from public or municipal databases, therefore highly reliable and with minimal loss to follow-up, recurrent ischemic event rates may have been underestimated due to the retrospective collection of clinical endpoints and possible recollection bias. Therefore, these results should be interpreted with caution and corroborated by future studies before making definitive conclusions.

Furthermore, it should be noted that in general, prognosis after STEMI has greatly improved over the last decades due to advanced care, e.g., revascularization strategies, highly effective antithrombotic medication and optimized infrastructure to minimize delay to treatment, as well as important advances in heart failure treatment and prevention of sudden cardiac death by implantable cardioverter-defibrillators in selected patients. One year mortality rates in the current era range from 2% in STEMI trials to ca. 7% in observational studies. In STEMI patients over 75 years of age, those who survived the first 30 days post STEMI had a long-term survival similar to the general population. A more recent large cohort study showed that excess mortality in all-comer first STEMI patients treated by primary PCI occurs mostly in the first 30 days, and that those who survived the first 90 days, had only 2% excess mortality at 10-years of follow-up compared to the general population [21]. The cause of death is predominantly cardiovascular in the first year after STEMI, but mainly non-cardiovascular thereafter [22,23]. In our multivariable analyses, only age remained associated with long-term outcome. Therefore, we hypothesize that in the current era, unlike early mortality after STEMI, long-term prognosis after STEMI is to a lesser extent determined by the index event and is increasingly determined by other factors, e.g., general atherosclerosis disease burden or progression, and non-cardiovascular co-morbidity, as indicated by the results of Fisher et al.[10]. Consequently, additional value of new treatment or risk-stratification strategies aimed at the index event may mostly lie in the first years after STEMI.

Several limitations should be considered. Cause of death was not recorded. Although the use of all-cause mortality is generally recommended and considered a strength, the differentiation between cardiovascular and non-cardiovascular death cause may provide more insight into the temporal relation of MVO with mortality. Clinical events other than death, i.e., recurrent MI or stroke, may have been underestimated due to recollection bias or selective loss to follow-up. Furthermore, data on ethnicity were not recorded due to legal restrictions. The study population was included over a long time span and although all patients underwent primary PCI, it cannot be excluded that long-term treatment strategies were heterogeneous as a consequence. Differences in long-term treatment, which were not recorded, may result in potential residual bias. Heart failure is a key component of clinical prognosis after STEMI, which is not reflected in the current study. Finally, the results of this study may not be representative for countries with scarce resources where urgent STEMI care is less readily available.

In conclusion, this study confirms a higher risk of mortality in those with MVO up to six years post-STEMI, yet this relation seems to dissipate thereafter. All other traditional prognosticators, except age, also lacked predictive value at long-term follow-up beyond six years. The prognostic implications of MVO were independent from cardiovascular risk factors, but not independent from infarct size and LV ejection fraction at long-term follow-up. Lastly, presence of MVO was not associated with recurrent ischemic events at long-term follow-up post-STEMI.

## Supporting information

S1 File The supporting file ‘MRI_prognosis_openshare.xlsx’ contains the clinical data underlying this study. (XLSX)

S1 Fig Central illustration. Abbreviations; MVO: Microvascular obstruction, STEMI: ST-segment elevation myocardial infarction.
(TIF)

## Abbreviations

CMR: cardiac magnetic resonance
LGE: late gadolinium enhancement
LV: left ventricular
MI: myocardial infarction
MVO: microvascular obstruction
PCI: percutaneous coronary intervention
STEMI: ST-segment elevation myocardial infarction
