# Supplementary figures and images for: Long-term prognostic value of microvascular obstruction by cardiac magnetic resonance in ST-segment elevation myocardial infarction
Source: PLoS One. 2026 Mar 6;21(3):e0344442. doi: 10.1371/journal.pone.0344442 (PMC12965695; doi:10.1371/journal.pone.0344442)

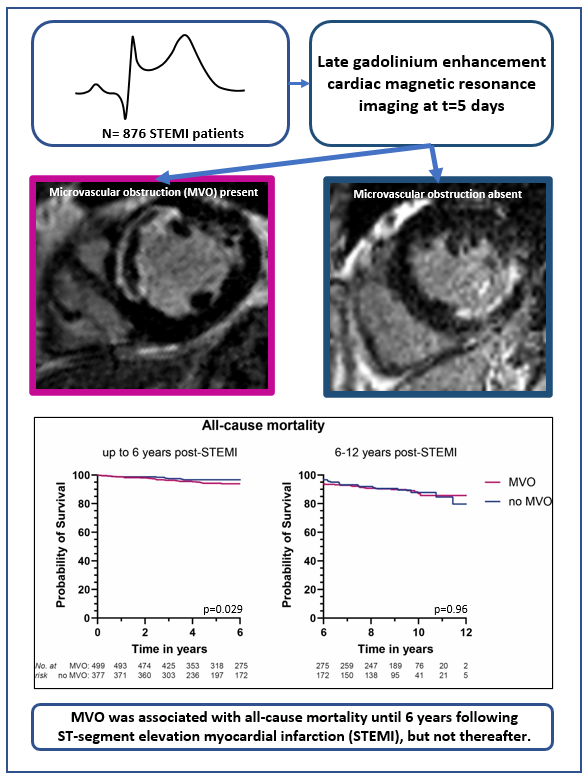

Supplement: S1 Fig — Abbreviations; MVO: Microvascular obstruction, STEMI: ST-segment elevation myocardial infarction. (TIF) [file pone.0344442.s002.tif]
